# Familiality of behavioral flexibility and response inhibition deficits in autism spectrum disorder (ASD)

**DOI:** 10.1186/s13229-019-0296-y

**Published:** 2019-12-12

**Authors:** Lauren M. Schmitt, Erin Bojanek, Stormi P. White, Michael E. Ragozzino, Edwin H. Cook, John A. Sweeney, Matthew W. Mosconi

**Affiliations:** 1 Division of Developmental and Behavioral Pediatrics, Cincinnati Children’s Hospital Medical Center, 3333 Burnet Ave., Cincinnati, OH 45229 USA; 2 Department of Psychiatry, University of Cincinnati College of Medicine, 260 Stetson St, Cincinnati, OH 45219 USA; 3 Schiefelbusch Institute for Life Span Studies/Clinical Child Psychology Program, University of Kansas, 1000 Sunnyside Ave., Lawrence, KS 66045 USA; 4 Kansas Center for Autism Research and Training, University of Kansas, 1000 Sunnyside Ave., Lawrence, KS 66045 USA; 5 Emory Department of Pediatrics, Marcus Autism Center, 1920 Briarcliff Rd NE, Atlanta, GA 30329 USA; 6 Department of Psychology, University of Illinois at Chicago, 1007 W Harrison St, Chicago, IL 60607 USA; 7 Institute for Juvenile Research, University of Illinois at Chicago, 1747 W Roosevelt Rd, Chicago, IL 60608 USA

## Abstract

**Background:**

Diminished cognitive control, including reduced behavioral flexibility and behavioral response inhibition, has been repeatedly documented in autism spectrum disorder (ASD). We evaluated behavioral flexibility and response inhibition in probands and their parents using a family trio design to determine the extent to which these cognitive control impairments represent familial traits associated with ASD.

**Methods:**

We examined 66 individuals with ASD (probands), 135 unaffected biological parents, and 76 typically developing controls. Participants completed a probabilistic reversal learning task (PRL) and a stop-signal task (SST) to assess behavioral flexibility and response inhibition respectively. Rates of PRL and SST errors were examined across groups, within families, and in relation to clinical and subclinical traits of ASD. Based on prior findings that subclinical broader autism phenotypic (BAP) traits may co-segregate within families and reflect heritable risk factors, we also examined whether cognitive control deficits were more prominent in families in which parents showed BAP features (BAP+).

**Results:**

Probands and parents each showed increased rates of PRL and SST errors relative to controls. Error rates across tasks were not related. SST error rates inter-correlated among probands and their parents. PRL errors were more severe in BAP+ parents and their children relative to BAP− parents and their children. For probands of BAP+ parents, PRL and SST error rates were associated with more severe social-communication abnormalities and repetitive behaviors, respectively.

**Conclusion:**

Reduced behavioral flexibility and response inhibition are present among probands and their unaffected parents, but represent unique familial deficits associated with ASD that track with separate clinical issues. Specifically, behavioral response inhibition impairments are familial in ASD and manifest independently from parental subclinical features. In contrast, behavioral flexibility deficits are selectively present in families with BAP characteristics, suggesting they co-segregate in families with parental subclinical social, communication, and rigid personality traits. Together, these findings provide evidence that behavioral flexibility and response inhibition impairments track differentially with ASD risk mechanisms and related behavioral traits.

## Introduction

Autism spectrum disorder (ASD) is a highly heritable neurodevelopmental disorder characterized by social-communication abnormalities and restricted, repetitive behaviors (RRBs). Numerous studies have documented the presence of a “broader autism phenotype” (BAP), a qualitatively similar, but milder, presentation of the defining ASD characteristics in some unaffected family members of individuals with ASD, suggesting inter-generational transmission of core ASD-related traits [1–3]. Yet, even with heritability estimates as high as 0.90 [4], our understanding of underlying pathophysiological processes and their relation to ASD traits remains limited owing, in part, to a lack of definitive biological and neurobehavioral markers of core clinical features [5]. Family studies identifying biologically based quantitative traits present in both individuals with ASD and their unaffected family members (i.e., endophenotypes) may help delineate characteristic patterns of inter-generational transmission and build mechanistic bridges between etiological processes and clinically relevant behavioral traits [6, 7].

Neurocognitive dimensions associated with core clinical features of ASD may represent important targets in this regard, as they are quantifiable and potentially more closely related to underlying neurobiological processes than broader clinical phenomena. Still, few studies have systematically examined neurocognitive traits in family members of individuals with ASD. Deficits in cognitive control have been repeatedly documented in individuals with ASD, and they have been linked to key clinical issues [8, 9]. Cognitive control is necessary for adaptive goal-directed behavior and includes neurobehavioral processes including behavioral flexibility (i.e., the ability to change behavior in response to contextual demands) and behavioral response inhibition (i.e., the ability to inhibit contextually inappropriate prepotent behaviors). Recent findings indicate that deficits in behavioral flexibility and response inhibition each uniquely contribute to higher-order RRBs, including insistence on sameness and compulsive behaviors [10], suggesting these cognitive control abilities represent distinct targets for family studies aimed at identifying endophenotypic markers associated with ASD.

Individuals with ASD show reduced behavioral flexibility characterized by an impaired ability to maintain new behavioral responses after previously reinforced responses are no longer rewarded [11–13]. They also show a reduced ability to withhold behavioral responses and use cognitive strategies to proactively delay response onset during tests of response inhibition [14, 15]. These cognitive control deficits are associated with more severe ASD symptoms including stereotyped speech and repetitive behaviors [11, 16, 17]. Thus, behavioral inflexibility may contribute to perseverative response patterns such as repetitive questioning despite attempts at redirection. Likewise, reduced response inhibition may contribute to patients seeking out strong interests even when these interests are contextually inappropriate.

Several studies have documented deficits in behavioral flexibility and response inhibition abilities in unaffected first-degree relatives of individuals with ASD, suggesting these deficits may serve as dimensional traits linked to familial risk [18–21]. Studies of these traits primarily have used traditional neuropsychological tests (e.g., Wisconsin Card Sorting Task, Stroop) that assess multiple cognitive processes simultaneously, making it difficult to determine the contributions of specific cognitive processes, especially for parents whose deficits are subclinical. Family trio studies examining inter-relationships of discrete cognitive control impairments among biological relatives of ASD probands can help determine whether these disorder-relevant impairments represent familial traits associated with ASD. This approach also can assess the extent to which cognitive control deficits covary with subclinical features in unaffected relatives to better understand inter-generational transmission of behavioral traits associated with ASD.

In the current study, we examined behavioral flexibility and behavioral response inhibition in probands with ASD and their biological mothers and fathers using tests that we previously validated in independent ASD samples [11, 15]. Based on our prior studies, we hypothesized that both probands and parents would show more errors than typically developing controls on tests of behavioral flexibility and response inhibition. Consistent with our hypothesis that specific cognitive control deficits represent separate neurodevelopmental risk pathways associated with ASD, we predicted that both behavioral flexibility and inhibition impairments would inter-correlate among probands and their parents, but that they would not inter-correlate with each other. To determine the extent to which cognitive control abilities tracked with core clinical and subclinical issues in probands and parents, behavioral flexibility and response inhibition were examined separately in families with BAP features (BAP+) and those without (BAP−). We predicted that reductions in behavioral flexibility and response inhibition would be greater for BAP+ parents and their offspring.

## Methods

### Participants

Forty-six family trios (including six multiplex families for which both affected siblings were examined) and 14 proband-parent dyads were studied. Twenty-nine parents whose child with ASD was unable to complete testing also were examined. Thus, a total of 66 probands and 135 parents were compared with separate groups of healthy controls matched on age, gender, and non-verbal IQ to probands (*n* = 29) and matched on gender and non-verbal IQ to parents (*n* = 47; Table 1). No parent controls were related to any of the proband controls. While the parent group was not age-matched to their control counterparts, age was not related to task performance in either the adult group (*r*’s < .19). Participant groups were matched on nonverbal rather than verbal IQ based on data indicating that individuals with ASD show less disorder-related weaknesses in nonverbal abilities [22]. Henceforth, controls matched to children and controls matched to parents will collectively be referred to as controls unless specified otherwise.

**Table 1:**
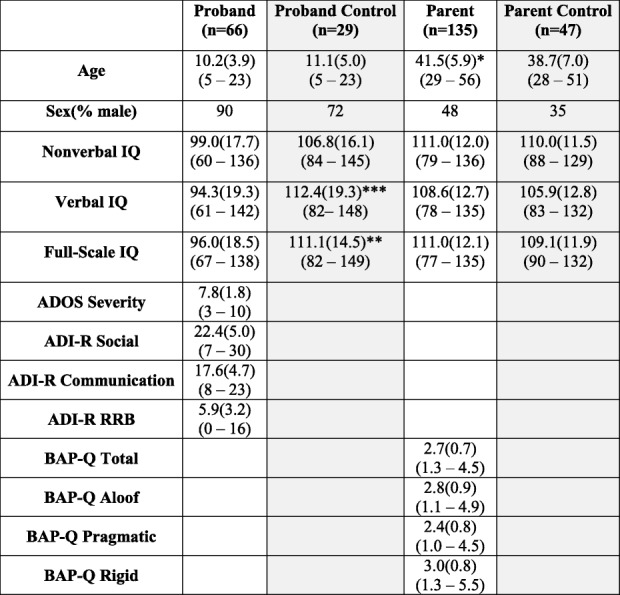
Participant demographic information

Mean (standard deviation), unless otherwise denoted. Range provided in parentheses on second line

Comparisons completed for corresponding group in gray (proband vs proband control; parent vs parent control), *p < .05, **p < .01, ***p < 0.001

Testing was conducted at the University of Illinois at Chicago (*n* = 39) and the University of Texas Southwestern (*n* = 238). Individuals with ASD and their parents were recruited through community advertisements and local outpatient clinics. ASD diagnoses were confirmed using the Autism Diagnostic Observation Schedule, 2nd Edition (ADOS-2 [23];), the Autism Diagnostic Interview-Revised (ADI-R [24];), and expert clinical opinion based on DSM-5 criteria [25]. Individuals with ASD were excluded if they had a known genetic disorder associated with ASD (e.g., Fragile X syndrome) or history of non-febrile seizures. All control participants were recruited through community advertisements and had a score of < 8 on the Social Communication Questionnaire (SCQ [26];). Controls were excluded if they had current or past psychiatric or neurological disorders, a family history of ASD in first- or second-degree relatives, or a history of developmental disorders or severe mental illness (e.g., schizophrenia) in first-degree relatives. Two parents with elevated SCQ scores completed the ADOS, but neither met the criteria for ASD. No participant had a history of head injury resulting in loss of consciousness. To ensure that the participants could understand all the task demands, only those with a NVIQ > 60 on the Wechsler Abbreviated Scale of Intelligence (WASI [27];) were included. Thirty-four participants (16 probands, 18 parents) were receiving psychotropic medication within 48 h of testing (Additional file 1). No controls were receiving any psychotropic medication within 4 weeks of participating in the study. When comparing probands/parents on-medication to those off-medication, groups did not differ on primary dependent variables (*p*’s > .16). Thus, all participants were included in our final analyses.

#### Ethics approval and consent to participate

All participants ≥ 18 years of age provided written consent, and minors provided assent and written consent was obtained from their legal guardians. Study procedures were approved by the University of Illinois at Chicago and the University of Texas Southwestern Institutional Review Boards.

### Procedure

#### Probabilistic reversal learning task

As described previously [11], during the probabilistic reversal learning task (PRL) task, participants were instructed to choose one of two identical stimuli (i.e., pictures of animals) positioned in different locations on the screen. Participants were reinforced (i.e., a coin appeared on the screen and placed into a money bag that kept track of total coins) on 80% of correct responses and on 20% of incorrect responses. During the acquisition phase, participants chose one of two stimulus locations until they identified the correct location on 8 of 10 consecutive trials. Then, they proceeded to the reversal phase in which the correct location was switched without warning, and participants had to identify the new correct location on 8 of 10 consecutive trials. Testing was discontinued if they did not reach the criterion within 50 trials during either phase. All participants completed two practice tests prior to PRL administration to establish test comprehension. Ten participants (7 probands, 1 parent, 2 controls) failed the acquisition phase, 14 participants (5 probands, 5 parents, 4 controls) failed the reversal phase, and 6 participants (4 probands, 4 parents) were not administered this test due to time constraints. Fifty probands, 125 parents, and 70 controls were included in the final analyses. We examined the number of errors (i.e., selecting the incorrect location) separately for acquisition and reversal phases.

#### Stop signal task

To examine behavioral inhibition, participants completed a stop-signal task (SST) consisting of interleaved GO and STOP trials as described previously [15, 28]. During GO trials, a target appeared to the left or right of the center, and participants responded as quickly as possible by pressing the button in the corresponding location. During STOP trials, a central STOP cue appeared at varying stop-signal delays (50–283 ms) after the GO cue, and participants were instructed to withhold their response. To ensure that participants did not delay their responses indefinitely, they received a prompt indicating “FASTER” and an “X” if they did not respond within 650 ms. The task consisted of 4 blocks of 63 trials (60% GO and 40% STOP trials) with similar ratio of GO to STOP trials in each of the 4 blocks. In order to ensure that each individual understood the instructions, participants completed a practice task consisting of 52 interleaved GO and STOP trials prior to the SST in which they had to demonstrate successful performance on 50% of STOP trials as done previously [15, 28].

Based upon our prior findings that probands show a reduced ability to proactively delay the onset of their responses and that increased slowing is associated with increased stopping success rate [15], baseline reaction times (RT) were measured during a task consisting of 60 GO trials administered prior to the SST. Three probands exceeded 650 ms average RT on > 50% of baseline trials and were not administered the SST. Additionally, five probands and one control failed to meet the practice criterion, and 19 individuals (8 probands, 11 controls) had scheduling issues that prevented completion of the SST. Fifty probands, 135 parents, and 64 controls were included in the final analyses. The proband participants who did not complete the SST were significantly younger than probands who completed the SST (*t* = 6.13, *p* < .001; mean age (SD) of non-completers 6.50 (2.2); mean age (SD) of completers 11.45 (3.9)). We computed the percentage of STOP trials in which participants inhibited their response and the difference in baseline GO and SST GO RTs. The order of tasks (PRL, SST) was randomly assigned to each participant.

#### Clinical measures

The ADI-R and ADOS were used to confirm clinical diagnoses and assess social-communication abnormalities and RRBs in probands. The ADI-R is a semi-structured caregiver interview used to characterize current and past clinical symptoms of ASD, including social abnormalities, communication impairments, and RRBs. The ADOS is a semi-structured assessment of social-communication impairments and RRBs. For each measure, higher scores represent more severe ASD symptoms.

In order to determine if PRL and SST performance covaried with subclinical ASD traits in parents of children with ASD, each parent completed the self-report version of the Broad Autism Phenotype-Questionnaire (BAP-Q [29];). The BAP-Q quantifies the severity of subclinical features of ASD, including social aloofness, pragmatic communication deficits, and rigid personality traits. As recently indicated, parental BAP is a useful tool to create phenotypically distinct subgroups of families of children with ASD [30]. Parents’ scores for each subdomain were compared against published norms [31]. As previously done [3], parents who scored above the identified BAP cutoffs on *any* subdomain were classified as “BAP+ parents,” and those who did not exceed any subdomain cutoff were categorized as “BAP− parents” (Table 2). Relative to prior studies, our sample demonstrated similar percentages of parents exceeding cutoffs for aloof (16%), pragmatic communication (25%), and rigid personality subscales (25%) or showing at least one BAP feature (~ 30% [2, 3, 32];). Probands with at least one BAP+ parent were categorized as “probands of BAP+ parents”; all other probands were categorized as “probands of BAP− parents.” Only two probands had both parents classified as BAP+ parents. Notably, probands of BAP+ parents and probands of BAP− parents did not differ on ADOS or ADI ratings (*p*’s > .22; Table 2). Ten parents did not complete the BAP-Q due to time constraints. Thirty-two BAP+ parents, 83 BAP− parents, 16 probands of BAP+ parents, and 30 probands of BAP− parents completed the PRL test. Thirty-four BAP+ parents, 91 BAP− parents, 18 probands of BAP+ parents, and 32 probands of BAP− parents completed the SST.

**Table 2:**
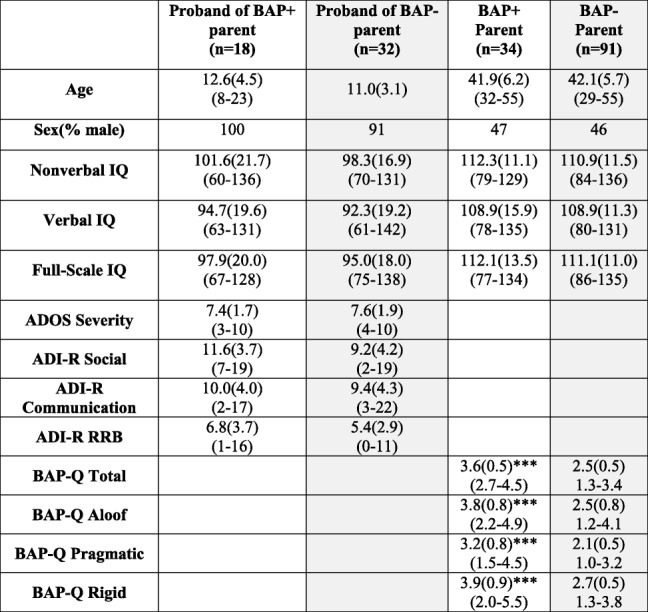
Demographic information based on BAP status

Mean (standard deviation), unless otherwise denoted. Range provided in parentheses on second line

Comparisons completed for corresponding group (gray), *p < .05, **p < .01, ***p < 0.001

### Statistical analyses

Each dependent variable was age-adjusted to account for non-linear relations between age and task performance as in previous work [33]. An inverse regression function was fit to data from healthy controls (combined proband control and parent control groups) from the current study to provide estimates of expected performance based on each participant’s age as in previous studies [34]. Then, the difference between each participant’s actual performance and their age-adjusted expected value was calculated, creating a deviation score for each participant for each dependent variable (Additional file 2). Deviation scores were converted to *Z*-scores based on the sample mean and standard deviation of all controls, with negative *Z*-scores denoting worse performance than expected given the individual’s age. For example, a negative *Z*-score for either PRL reversal phase errors or SST errors would indicate the participant is making *more* errors than would be expected given their chronological age, and a negative *Z*-score for SST RT slowing would reflect *reduced* RT slowing than expected. Distributions for each of our primary cognitive control outcomes for each subgroup are shown in Additional file 4.

Separate ANOVAs were used to examine each age-adjusted dependent variable (*Z*-score) with group (proband vs parent vs control) as the between-subject factor. Significant effects were probed with planned pairwise comparisons using Bonferroni corrections for multiple comparisons. Due to possible impact of including six multiplex families, we removed one proband from each of these families (at random) and conducted all the analyses a second time. Results were not substantively different, so all probands were included in the final analyses. PRL reversal phase errors were not normally distributed (kurtosis: proband = 1.235; parent = 4.465; control = 6.269), so non-parametric Kruskal-Wallis *H* tests were conducted.

In order to determine whether parents demonstrating subclinical ASD features and their offspring demonstrated greater cognitive control issues than controls, we conducted separate ANOVAs comparing probands of BAP+ parents, probands of BAP− parents, and controls as well as BAP+ parents, BAP− parents, and controls on each dependent variable. Initial analyses of individual tasks included any participant who completed that task. Follow-up analyses including only the subset of individuals who completed all tasks were not substantively different (proband = 39, parent = 125, control = 57; Additional file 3).

To estimate the familiality of behavioral flexibility and inhibition deficits in family trios, Sequential Oligogenetic Linkage Analysis Routines (SOLAR) was used [35]. This analysis approach provides estimates of familiality (*h*^2^) representing the proportion of variance in PRL or SST performance accounted for by family membership. Maximum likelihood estimates were used to compare a model in which performance is explained by family membership relative to a model in which family membership is not considered.

In order to examine inter-relationships between behavioral flexibility and response inhibition in probands, parents, and controls, separate non-parametric Spearman correlations were conducted for each group. For probands only, we examined the relationships between behavioral flexibility and inhibition deficits with ADI-R (ADI-R [24];) and ADOS-2 (ADOS-2 [23];) ratings of ASD symptoms. The revised algorithms for modules 1–3 [36] and module 4 [37] were used. In order to determine whether deficits are more severe for probands who have parents with subclinical traits, we conducted the same analyses separately for probands of BAP+ parents and probands of BAP− parents. To reduce type I error rates, we only considered relationships significant if |*r*| > .50 or *p* < .01.

## Results

**Fig. 1:**
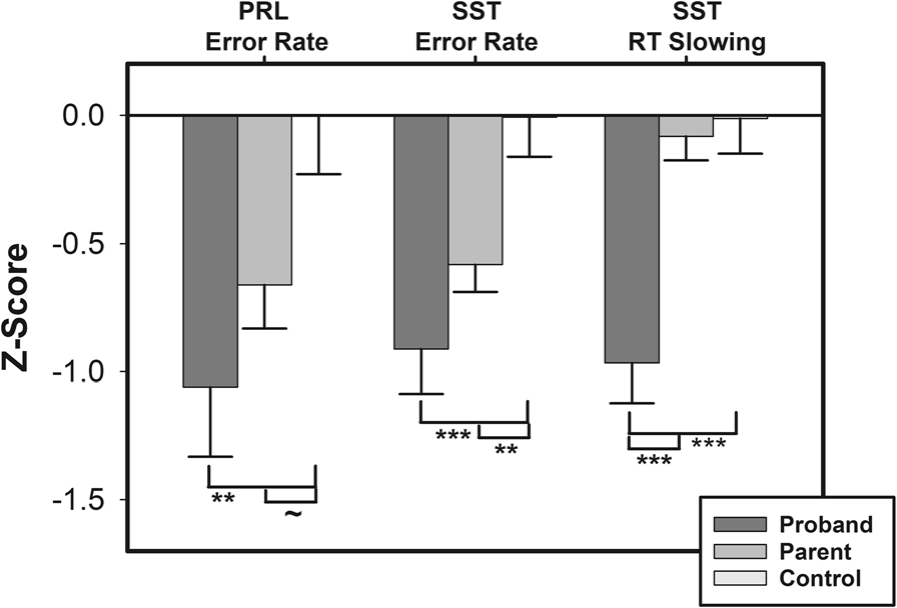
Behavioral flexibility and inhibitory control in individuals with ASD (proband), parents of individuals with ASD, and controls. PRL error rate, SST error rate, and SST reaction time slowing were significantly worse in ASD probands compared to controls. Parents of individuals with ASD also show significantly worse SST error rate than controls, but PRL error rate only trended towards significance. Negative Z-scores denote worse performance (e.g., higher error rate or reduced slowing). Error bars represent standard error. ~*p* < 0.07, **p < 0.01, ***p < 0.001. PRL, probabilistic reversal learning; SST, stop-signal task; RT, reaction time

During the PRL acquisition phase, there was no difference in the number of errors between probands, parents, and controls (*F* (2, 256) = .93, *p* = .40). However, proband, parent, and control groups differed on the number of errors during the PRL reversal phase (Table 3; Fig. 1; *Χ*^2^(2) = 7.931, *p* = .02), on the percentage of STOP trial errors made during the SST (Fig. 1; *F* (2, 245) = 8.19, *p* < .001, *η*^2^_p_ = .06), and on the amount of RT slowing from baseline to SST GO trials (Fig. 1; (*F* (2, 245) = 13.60, *p* < .001, *η*^2^_p_ = .10). Individuals with ASD made more reversal phase PRL errors than controls (*p* = .03), but not parents (*p* = .79). Parents made more errors than controls (*p* = .01). During the SST, probands (*t* (112) = − 3.89, *p* < .001) and parents (*p* = .002) each made more STOP errors than controls, but probands and parents did not differ from each other (*p* = .11). During the SST, probands also showed less RT slowing than controls (*p* < .001) and parents (*p* < .001), but parents and controls demonstrated similar levels of RT slowing (*p* = .67).

**Table 3:** ANOVA results from comparisons of probands, parents, and healthy control participants on probabilistic reversal learning (PRL) and stop-signal task (SST)

		df_n_, df_d_	*F*	*p*	*η*^2^_p_	Post hoc	*p*
PRL	Acquisition errors	2, 256	0.93	0.40	0.007	–	
	Reversal errors	2, 246	4.82	0.01	0.04	Proband vs control	0.01
						Parent vs control	0.06
						Proband vs parent	0.35
SST	STOP trial errors	2, 245	8.19	< .001	0.06	Proband vs control	< .001
						Parent vs control	< .001
						Proband vs parent	0.11
	RT slowing	2, 245	13.60	< .001	0.10	Proband vs control	< .001
						Parent vs control	< .001
						Proband vs parent	0.67

*df*_*n*_ degrees of freedom numerator, *df*_*d*_ degrees of freedom denominator, *η*^*2*^_*p*_ partial eta-squared

### Familiality of behavioral flexibility and inhibition deficits

STOP trial error rates were significantly familial (Table 4; *h*^2^ = .54, *p* = .007). However, neither the number of PRL reversal errors made (*h*^2^ < .001, *p* = .500) nor SST RT slowing (*h*^2^ = .079, *p* = .334) were familial.

**Table 4:** Familiality estimates using SOLAR

	*h*^2^	*p*
PRL error rate	< .001	.500
SST error rate	.54	.007
SST RT slowing	.08	.334

### Associations between cognitive control and BAP traits

**Fig. 2:**
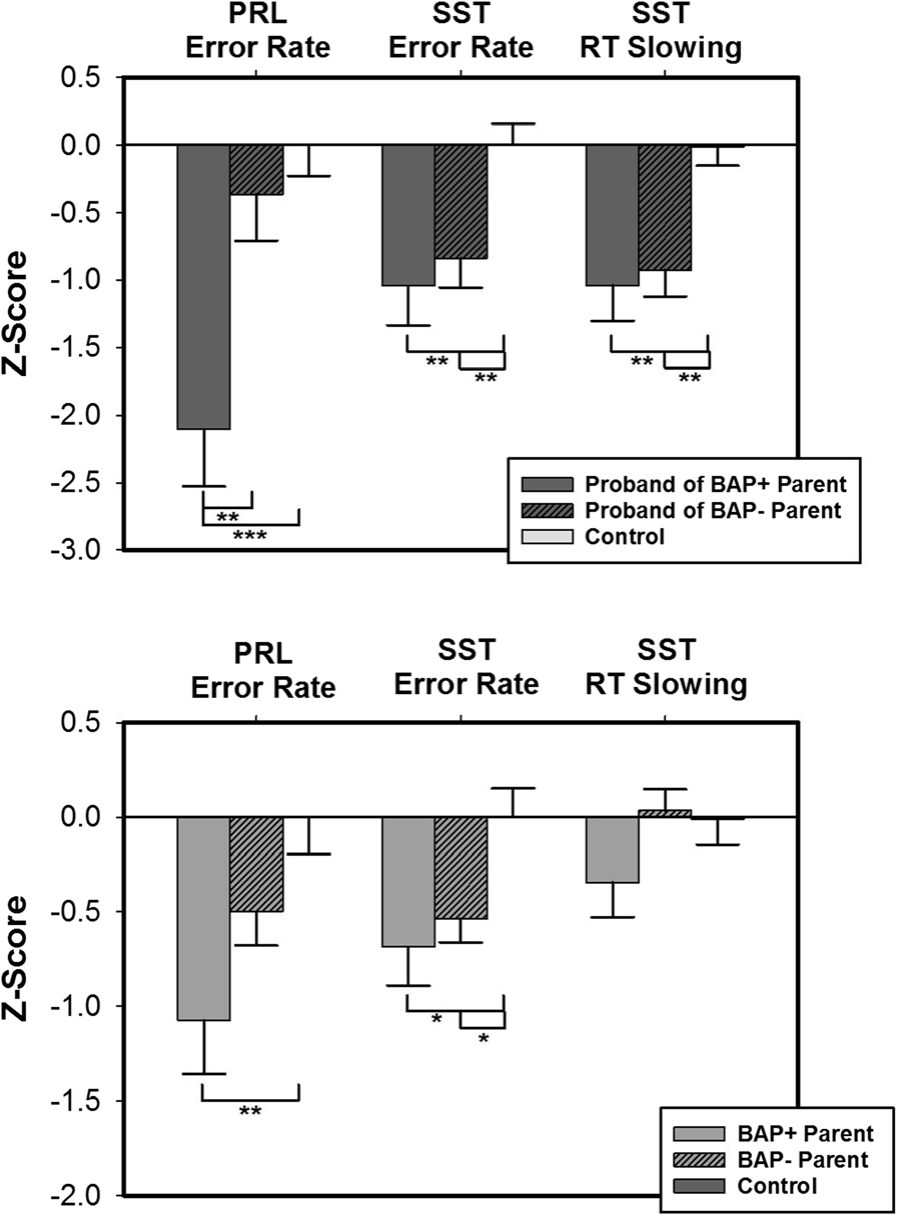
Behavioral flexibility and inhibitory control impairments in probands and parents based on BAP status. Among probands of BAP+ parents, PRL error rate, SST error rate, and SST reaction time slowing were significantly worse than controls. PRL error rate also was significantly increased between probands of BAP+ parents and probands of BAP− parents (top). Among BAP+ parents, PRL error rate and SST error rate were significantly greater than controls. Among BAP− parents, SST error rate also was significantly greater than controls (bottom). Negative *Z*-scores denote worse performance (e.g., higher error rate or reduced slowing). Error bars represent standard error. *p < 0.05, **p < 0.01, ***p < 0.001. PRL, probabilistic reversal learning; SST, stop-signal task; RT, reaction time; BAP+, presence of broad autism phenotype features; BAP−, absence of broad autism phenotype features

Probands of BAP+ parents, probands of BAP− parents, and controls differed on the number of errors made during the PRL reversal phase (Table 5; Fig. 2; *Χ*^2^(2) = 6.95, *p* = .03), the rate of errors during the SST (Fig. 2; *F* (2, 110) = 7.70, *p* = .001, *η*^2^_p_ = .12), and the amount of their RT slowing during the SST (Fig. 2; *F* (2, 110) = 5.48, *p* < .001, *η*^2^_*p*_ = .09). Probands of BAP+ parents made more PRL reversal errors than controls (*p* = .03) and probands of BAP− parents, though this effect was not significant (*p* = .09). Probands of BAP− parents did not differ from controls (*p* = .99) on PRL reversal phase errors. Probands of both BAP+ (*p* = .01) and BAP− parents (*p* = .01) made more SST STOP errors than controls. Similarly, probands of BAP+ parents (*p* = .002) and probands of BAP− parents (*p* = .001) showed reduced RT slowing compared to controls, but probands of BAP+ parents and probands of BAP− parents did not differ from each other (*p* = .57).

**Table 5:** ANOVA results from comparisons of probands of BAP+ parents, probands of BAP− parents, and healthy control participants on probabilistic reversal learning (PRL) and stop-signal task (SST)

		df_n_, df_d_	F	*p*	η^2^_p_	Post-Hoc	*p*
PRL	Acquisition errors	2, 117	0.32	0.73	0.005	–	
	Reversal errors	2, 117	9.67	< .001	0.14	Proband of BAP+ parent vs control	< .001
						Proband of BAP− parent vs control	0.99
						Proband of BAP+ parent vs of BAP− parent	0.006
SST	STOP trial errors	2, 110	7.70	< .001	0.12	Proband of BAP+ parent vs control	0.01
						Proband of BAP- parent vs control	0.01
						Proband of BAP+ parent vs of BAP− parent	0.11
	RT slowing	2, 110	5.48	< .001	0.09	Proband of BAP+ parent vs control	< .001
						Proband of BAP- parent vs control	.001
						Proband of BAP+ parent vs of BAP− parent	0.57

*df*_*n*_ degrees of freedom numerator, *df*_*d*_ degrees of freedom denominator, *η*^*2*^_*p*_ partial eta-squared

BAP+ parents, BAP− parents, and controls differed in their PRL reversal phase error rates (Table 6; Fig. 2; *Χ*^2^(2) = 6.122, *p* = .04) and in their SST STOP trial error rates (Fig. 2; *F* (2, 188) = 5.11, *p* = .01, *η*^2^_p_ = .05). BAP+ parents made marginally more PRL reversal phase errors than controls (*p* = .06); however, BAP− parents did not differ from controls (*p* = .24) or BAP+ parents (*p* = .95). On the SST, BAP+ parents (*p* = .02) and BAP− parents (*p* = .02) each made more STOP errors than controls, and BAP+ and BAP− parents did not differ from each other (*p* = .99). RT slowing did not differ between BAP+ parents, BAP− parents, and controls (Fig. 2; *F* (2, 188) = 1.62, *p* = .20, *η*^2^_p_ = .02).

**Table 6:** ANOVA results from comparisons of BAP+ parents, BAP- parents, and healthy control participants on probabilistic reversal learning (PRL) and stop-signal task (SST)

		df_n_, df_d_	*F*	*p*	*η*^2^_p_	Post hoc	*p*
PRL	Acquisition errors	2, 183	0.81	0.45	.009	–	
	Reversal errors	2, 183	5.08	.01	.007	BAP+ parent vs control	0.01
						BAP− parent vs control	0.18
						BAP+ parent vs BAP− parent	0.26
SST	STOP trial errors	2, 188	5.11	.01	0.05	BAP+ parent vs control	0.02
						BAP− parent vs control	0.02
						BAP+ parent vs BAP− parent	0.99
	RT slowing	2, 188	1.62	0.20	0.02	–	

*df*_*n*_ degrees of freedom numerator, *df*_*d*_ degrees of freedom denominator, *η*^*2*^_*p*_ partial eta-squared

### Associations between cognitive control and clinical deficits

PRL and SST performance was not associated with IQ for any group (|*r*|’s < .38). Greater RT slowing was associated with fewer SST errors for all groups (ASD: *r* = .45, *p* = .001; parent: *r* = .48, *p* < .001; control: *r* = .46, *p* < .001). PRL errors were not associated with SST errors or slowing for probands, parents, or controls (|*r*|’s < .13). Similarly, PRL errors were not associated with SST errors or slowing for probands of BAP+ parents, probands of BAP− parents, BAP+ parents, or BAP− parents, *p* (|*r*|’s < .12).

For probands of BAP+ parents, more PRL errors were associated with more severe ADI-rated communication abnormalities (*r* = − .70, *p* = .005). Greater SST error rates (*r* = − .50, *p* = .05) and reduced RT slowing (*r* = − .52, *p* = .04) each were associated with more severe ADI-rated higher-order repetitive behaviors (algorithm items C1+C2), but not lower-order RRBs (algorithm items C3+C4; |*r*|’s < .32). No significant associations were observed between cognitive control abilities and clinical issues for probands of BAP− parents or the overall proband group (|*r*|’s < .35).

## Discussion

In the present study, we document three key findings regarding cognitive control deficits in ASD. First, behavioral flexibility and response inhibition abilities were impaired in both individuals with ASD and their unaffected biological parents. Importantly, behavioral flexibility and response inhibition abilities were not related to each other and each was associated with separate core ASD symptoms. Second, a reduced ability to inhibit prepotent behavioral responses inter-correlated among individuals with ASD and their parents, suggesting reduced inhibitory control is a familial neurocognitive trait in ASD. To our knowledge, this is the first study to document the inter-correlation of a neurocognitive trait among individuals with ASD and their unaffected biological parents, suggesting behavioral response inhibition may represent an important endophenotype in this neurodevelopmental disorder. Third, reductions in behavioral flexibility were more profound in BAP+ parents and their children with ASD, indicating behavioral flexibility may be selectively affected in a subset of ASD families in which subclinical social, communication, or rigid personality traits are present. Together, our findings provide novel evidence that behavioral flexibility and response inhibition represent separate familial trait dimensions that each may be an important associated risk marker for ASD.

### Cognitive control impairments in individuals with ASD

Our results from the PRL test confirm that individuals with ASD demonstrate an impaired ability to switch to and maintain new behavioral responses when a previously reinforced response is no longer contextually appropriate, especially among probands of BAP+ parents [11, 12, 38]. Findings from the SST also confirm that individuals with ASD have deficits withholding prepotent behavioral responses and implementing proactive strategies to determine the contextual appropriateness of their behavioral responses regardless of the presence of BAP features in their parents [14, 15, 18]. Importantly, we extend prior studies by demonstrating that behavioral flexibility and response inhibition deficits are not related to one another in individuals with ASD, consistent with findings in typically developing controls [39]. The inference that behavioral flexibility and response inhibition deficits are distinct from one another also is supported by our findings that each is associated with separate clinically rated ASD symptoms. Among probands of BAP+ parents, difficulties switching to and maintaining new behavioral response preferences during the PRL task were associated with more severe social-communication impairments. This expands upon our previous finding of a relationship between reduced behavioral flexibility and more severe stereotyped speech in ASD by suggesting that failures to switch away from preferred behavioral responses and maintain new ones may relate more broadly to social-communication abnormalities in patients [11]. Thus, it is possible that failures to flexibly shift behavioral responses in response to new reward contingencies may interfere with the ability to adapt social-communication strategies to different environmental demands.

In contrast, reduced abilities to inhibit and delay prepotent responses during the SST were selectively associated with more severe higher-order RRBs, but not repetitive sensorimotor behaviors, as our group and others have previously documented [10, 15–17, 40]. Thus, failures to suppress contextually inappropriate behaviors may interfere with the ability to refrain from completing highly ritualized or preferred behaviors or seeking out intense interests [10, 15, 16, 41]. Likewise, diminished preparatory control of behavior may interfere with adapting to unpredictable changes in the environment or in routines. Though results from the present study indicate that the distinct relationships between cognitive control deficits and core ASD features only were significant for probands of BAP+ parents, ASD symptom severity was similar across patient subgroups, suggesting that these relationships were not simply a product of probands of BAP+ parents being more severely affected. Instead, our findings suggest that these traits are more likely to covary in a select subgroup of patients whose parents display subclinical ASD features. Together, our results provide evidence that the neurocognitive processes underlying deficits in behavioral flexibility and response inhibition track separately with distinct sets of clinical correlates, and thus may reflect distinct risk pathways in ASD.

### Cognitive control in parents of individuals with ASD

Our results show that behavioral flexibility and response inhibition ability are reduced in both probands and their parents and, importantly, that difficulty inhibiting prepotent responses is familial in ASD. While previous studies indicate the presence of a broader range of subclinical characteristics associated with ASD in unaffected parents than BAP traits alone (i.e., psychiatric, sensorimotor, and neuroanatomical features [18, 20, 42, 43];), this is the first known study to document the inter-correlation of a neurocognitive trait among individuals with ASD and their unaffected biological parents. This finding provides novel evidence that reductions in the ability to inhibit behavioral responses reflect a quantifiable dimension of inter-generational risk for ASD. It is possible that the familiality of response inhibition deficits reflects a process in which probands with ASD model traits from their parents, though twin studies previously have suggested that behavioral response inhibition is highly heritable (heritability estimates = 0.50 [44];). Twin studies of behavioral response inhibition in affected and unaffected siblings will be important for parsing the heritability of inhibitory control deficits in ASD, but our findings provide new evidence that the high levels of heritability and complex genetic architecture of ASD may reflect an inheritance of distinct risks for illness identifiable by neurocognitive trait markers in select families.

We also found reductions in behavioral flexibility among parents of individuals with ASD, and pair-wise comparisons indicated that BAP+ parents showed marginal but non-significant reductions in their ability to flexibly shift behavior away from a previously rewarded response pattern relative to controls, whereas no effect was seen for BAP− parents. These findings implicate behavioral inflexibility as familial in BAP+ families, though error rates were not normally distributed indicating the familiality of behavioral inflexbility may reflect liability in a select subgroup of BAP+ parents. It also suggests behavioral inflexibility may be part of a broader constellation of BAP traits in these families. Of note, we found that probands of BAP+ parents demonstrated greater behavioral flexibility impairments than probands of BAP− parents, suggesting that behavioral inflexibility in patients may systemically vary based on the presence of parental subclinical traits (Additional files 5 and 6).

The etiological heterogeneity in ASD is well-documented (for examples, see [45–47]), and there exists an urgent need to identify more homogeneous subgroups of ASD based on co-segregation of pathophysiological processes or behavioral phenotypes. Our results indicate that behavioral flexibility issues and BAP features co-segregate and may represent a biologically distinct cluster of families with affected children. Importantly, measures of behavioral flexibility are highly objective and thus provide powerful tools for quantifying familial risk or characterizing discrete family clusters. Further, prior studies documenting relationships between behavioral inflexibility and atypical brain activation in prefrontal cortex, motor cortex, parietal cortex, and dorsal striatum in ASD implicate discrete neural networks that serve as key targets for determining neurobiological endophenotypes [12]. Thus, our findings suggest that familial trait dimensions extend to neurocognitive traits, providing evidence that distinct etiological pathways, including disruptions to fronto-parietal-striatal circuitry, may differentially characterize BAP+ and BAP− families.

Our results demonstrate that behavioral flexibility and response inhibition each may represent familial traits related to ASD risk. Still, their validity as endophenotypes according to the criteria laid out by Gottesman and Gould’s [7] can be questioned based on our finding that the severity of behavioral flexibility and inhibitory control issues did not differ between probands and parents as would be expected given an additive risk factor model (i.e., probands < parents < controls). However, direct comparison of effect size of deficits in adults and children is complicated due to heterogeneity in cognitive development trajectories that increases variance in cognitive measures. It also is possible that our findings are only evident when adjusting performance for age as this allows us to detect deviations from normative developmental trajectories for neurocognitive processes that control to mature into adulthood ([34]; D’Cruz 2016). Indeed, exploratory analyses (Additional file 6) of raw data without adjustments for age indicate PRL error rate and SST error rate are significantly higher among probands compared to parents when age is not accounted for. Given the maturation of cognitive flexibility and behavioral inhibition into late adolescence and early adulthood, even among individuals with ASD, this finding is not surprising. Further, it strengthens our finding in parents of individuals with ASD by demonstrating degree of neurocognitive deficits is similar to probands once age is accounted for. Additionally, these neurocognitive traits may not follow a traditional additive risk model, such that behavioral flexibility and response inhibition deficits may reflect familial traits that influence ASD phenotypes superimposed upon other disorder-related liabilities (e.g., deficits of attention or sensorimotor control) to magnify their expression (“BASINS” [48, 49];). This hypothesis suggests that cognitive control deficits may not be specific to ASD, but that their presence in addition to other traits may influence the clinical manifestations of ASD (e.g., ADHD, OCD; for examples, see [50, 51]).

### Limitations

There are certain limitations of the present study. First, given that behavioral flexibility and response inhibition deficits also are seen in other neuropsychiatric disorders, including ADHD, it will be important to examine their relation to other trait dimensions or clinical issues in ASD. Second, while our study relied on experimental tasks that we have previously validated in individuals with ASD, it will be important to examine their association with additional measures of cognitive control and separate neurocognitive functions implicated in ASD to capture latent constructs that better characterize distinct familial mechanisms [10]. Third, despite our relatively large sample size, larger family trio studies are needed to determine the extent to which errors in behavioral flexibility and response inhibition reflect independent relationships with specific BAP features, and whether familiality differs in multiplex versus simplex families and mother-proband versus father-proband dyads. Additionally, larger family trio samples are needed to evaluate a greater number of probands of BAP+ parents. Last, we used healthy controls matched to probands and parents that were not related to each other, which may have biased findings. Thus, future studies are needed using healthy control children and their two biological parents.

## Conclusion

Our study provides new evidence that behavioral flexibility and response inhibition deficits represent discrete familial traits in ASD. Our findings that separate neurocognitive dimensions associated with ASD track in different families and with different symptom clusters indicate that these traits may provide important markers of distinct neurobehavioral alterations associated with ASD. Identifying neurocognitive trait dimensions in ASD families is a promising strategy for better understanding distinct pathophysiological processes and potential neurodevelopmental risk pathways in ASD that may be useful in parsing etiological heterogeneity as has been done successfully in studies of other neuropsychiatric disorders [6].

## Supplementary information

**Additional file 1:** Medication type by subject type.

**Additional file 2:** Raw scores for ASD probands and parents (black circle) and controls (open square).

**Additional file 3.** Results for participants who completed both PRL and SST tasks.

**Additional file 4:** Histograms of primary variables (z-scores, negative value indicating worse performance) for Probands, Parents, and Controls (collapsed).

**Additional file 5.** Correlation Matrix of Relationship between BAP-Q scores and Primary Cognitive Control Variables for Parents of Individuals with ASD.

**Additional file 6.** Non-age adjusted findings.

## Data Availability

Data and materials are available upon direct request to the corresponding author.

## Abbreviations

ADHD: Attention-deficit/hyperactivity disorder
ADI-R: Autism Diagnostic Interview-Revised
ADOS: Autism Diagnostic Observation Schedule
ANOVA: Analysis of variance
ASD: Autism spectrum disorder
BAP: Broad autism phenotype
BAP−: Parent of individual with ASD who does not demonstrate BAP features
BAP+: Parent of individual with ASD who demonstrates BAP feature(s)
BAP-Q: Broad Autism Phenotype Questionnaire
IQ: Intelligence quotient
MRI: Magnetic resonance imaging
OCD: Obsessive-compulsive disorder
PRL: Probabilistic reversal learning
RRB: Restricted, repetitive behaviors
RT: Reaction time
SCQ: Social Communication Questionnaire
SD: Standard deviation
SOLAR: Sequential Oligogenetic Linkage Analysis Routines
SST: Stop-signal task
WASI: Wechsler Abbreviated Scale of Intelligence
