# Supplementary material for: Familiality of behavioral flexibility and response inhibition deficits in autism spectrum disorder (ASD)
Source: Mol Autism. 2019 Dec 12;10:47. doi: 10.1186/s13229-019-0296-y (PMC6909569; doi:10.1186/s13229-019-0296-y)
Supplement: Supplementary file 1 — Additional file 1: Medication type by subject type. [file 13229_2019_296_MOESM1_ESM.docx]

Additional File 1. Medication type by subject type.

| Subject Type | AA | AD | MS | SH | ST |
| --- | --- | --- | --- | --- | --- |
| proband | 1 | 1 |  |  | 1 |
| proband | 1 | 1 |  |  |  |
| proband | 1 |  |  |  |  |
| proband |  | 1 |  |  | 1 |
| proband |  | 1 |  |  | 1 |
| proband |  | 1 |  |  | 1 |
| proband |  | 1 |  |  |  |
| proband |  | 1 |  |  |  |
| proband |  | 1 |  |  |  |
| proband |  | 1 |  |  |  |
| proband |  |  |  | 1 | 1 |
| proband |  |  |  |  | 1 |
| proband |  |  |  |  | 1 |
| proband |  |  |  |  | 1 |
| proband |  |  |  |  | 1 |
| proband |  |  |  |  | 1 |
| proband |  |  |  |  | 1 |
| parent | 1 | 1 |  |  |  |
| parent |  | 1 |  |  |  |
| parent |  | 1 |  |  |  |
| parent |  | 1 | 1 | 1 |  |
| parent |  | 1 |  | 1 |  |
| parent |  | 1 |  | 1 |  |
| parent |  | 1 |  |  |  |
| parent |  | 1 |  |  |  |
| parent |  | 1 |  |  |  |
| parent |  | 1 |  |  |  |
| parent |  | 1 |  |  |  |
| parent |  | 1 |  |  |  |
| parent |  | 1 |  |  |  |
| parent |  | 1 |  |  |  |
| parent |  |  |  |  | 1 |
| parent |  |  |  | 1 |  |
| parent |  |  |  | 1 |  |
| parent |  |  |  |  | 1 |

1 indicates the participant was taking medication within 48 hours of testing, empty cell indicates no medication.

AA=atypical antipsychotic; AD=anti-depressant; MS=mood stabilizer; SH=sedative, hypnotic, anxiolytic; ST=stimulant
