# Supplementary material for: Familiality of behavioral flexibility and response inhibition deficits in autism spectrum disorder (ASD)
Source: Mol Autism. 2019 Dec 12;10:47. doi: 10.1186/s13229-019-0296-y (PMC6909569; doi:10.1186/s13229-019-0296-y)
Supplement: Supplementary file 2 — Additional file 2: Raw scores for ASD probands and parents (black circle) and controls (open square). [file 13229_2019_296_MOESM2_ESM.docx]

Additional file 2. Raw scores for ASD probands and parents (black circle) and controls (open square).

Deviation score calculated by subtracting raw value from expected value, with a larger and more negative value indicating worse performance

}
