# Supplementary material for: Familiality of behavioral flexibility and response inhibition deficits in autism spectrum disorder (ASD)
Source: Mol Autism. 2019 Dec 12;10:47. doi: 10.1186/s13229-019-0296-y (PMC6909569; doi:10.1186/s13229-019-0296-y)
Supplement: Supplementary file 3 — Additional file 3. Results for participants who completed both PRL and SST tasks. [file 13229_2019_296_MOESM3_ESM.docx]

Additional Information 3. Results for participants who completed both PRL and SST tasks

When examining participants who completed both PRL and SST tests, there was no longer an overall effect of group in PRL error rate (F(2,218)=2.836, p=.061). However, when examining proband and parent performance compared to control performance separately, both individuals with ASD (t(95)=-1.971, p=.049) and parents (t(181)=-2.181, p=.031) had increased PRL error rate relative to controls. During the SST, accuracy on STOP trials (F(2,218)=5.964, p=.003) and RT slowing (F(2,218)=9.593, p<.001) were different between groups. Probands (t(95)=-3.109, p=.006) and parents (t(181)=-2.919, p=.012) had reduced STOP accuracy compared to controls, but probands did not differ from parents (t(163)=-.978, p=.987). Probands had reduced latency slowing compared to controls (t(95)=-3.716, p=.001) and parents (t(163)=-4.203, p<.001), but parents did not differ from controls (t(181)=-.011, p=.999).
