# Supplementary material for: Familiality of behavioral flexibility and response inhibition deficits in autism spectrum disorder (ASD)
Source: Mol Autism. 2019 Dec 12;10:47. doi: 10.1186/s13229-019-0296-y (PMC6909569; doi:10.1186/s13229-019-0296-y)

Additional file 4. Histograms of primary variables (z-scores, negative value indicating worse performance) for Probands, Parents, and Controls (collapsed).

**Controlss**

**Parentss**

**Probands**


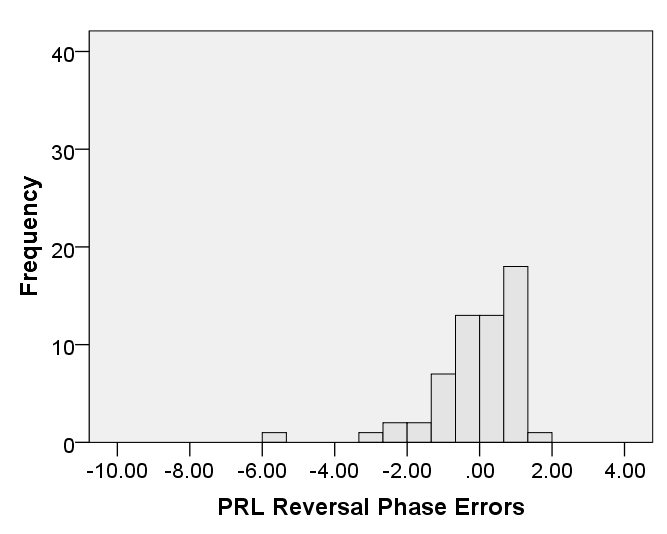


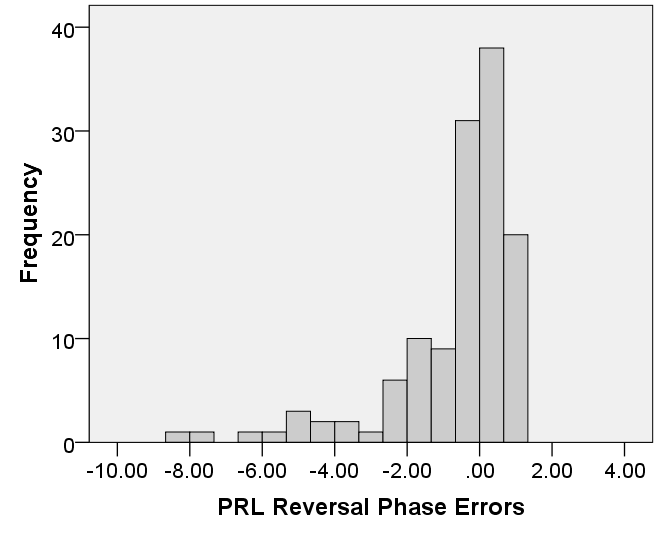

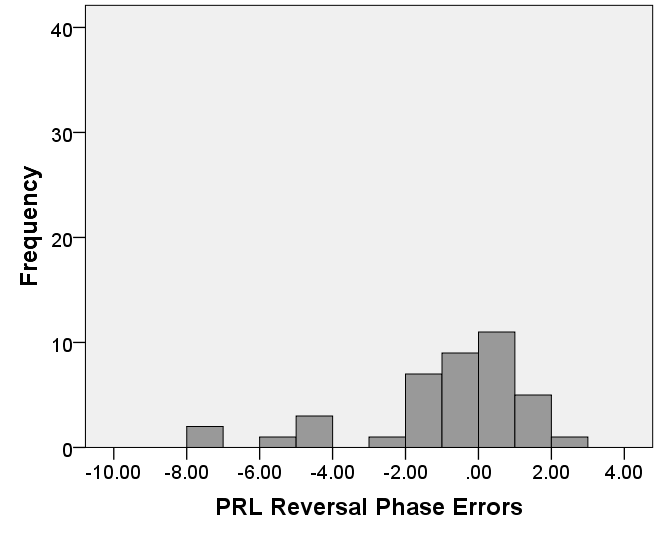


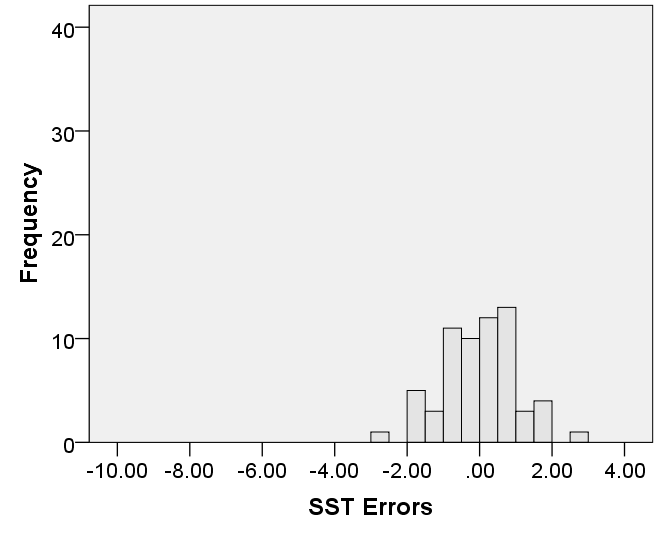

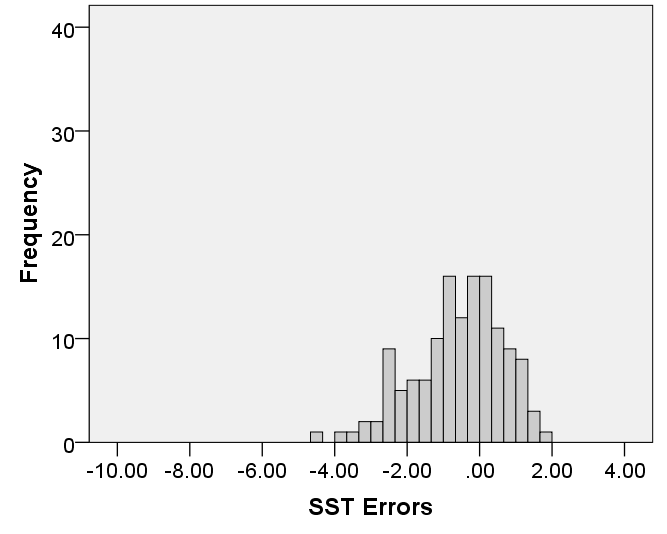

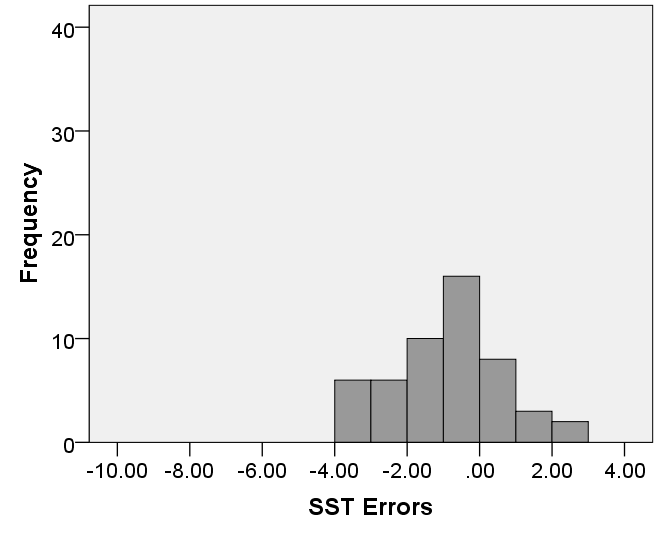


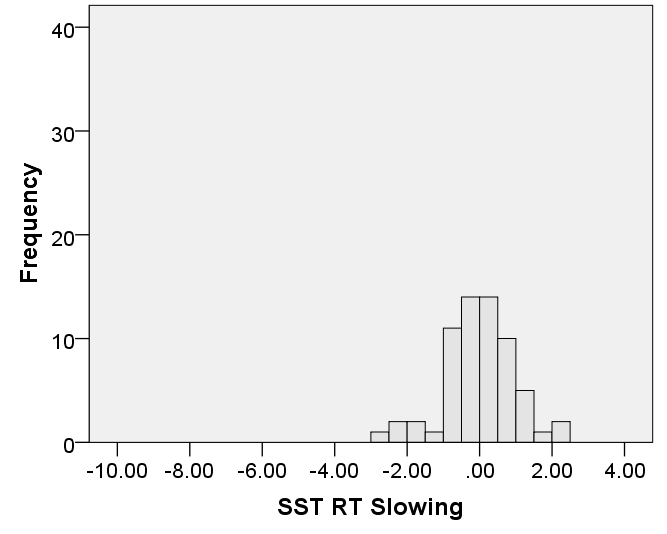

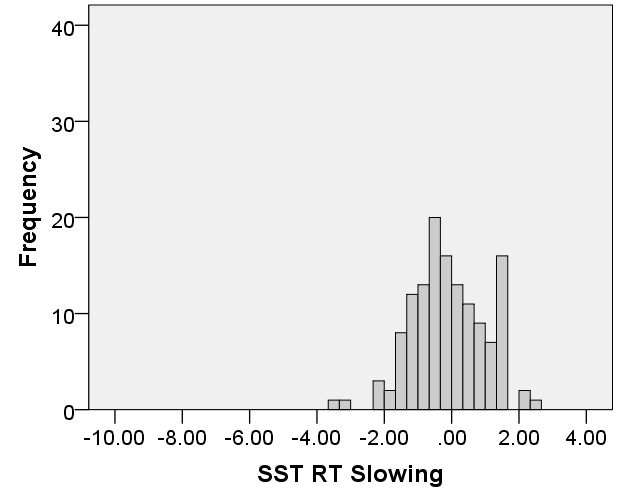

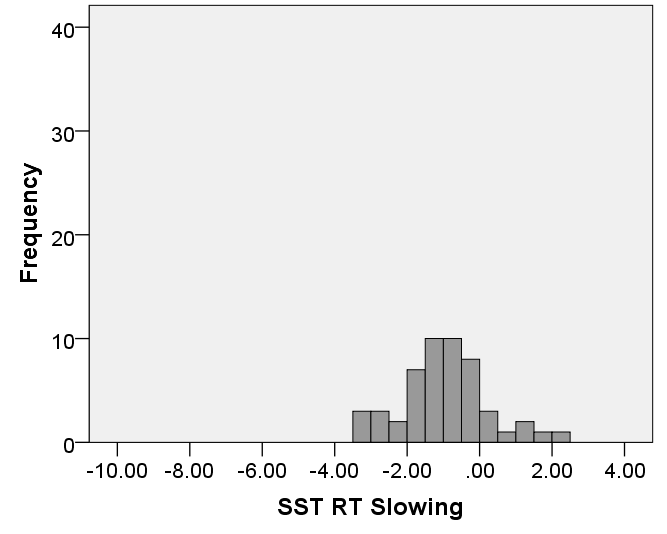

Supplement: Supplementary file 4 — Additional file 4: Histograms of primary variables (z-scores, negative value indicating worse performance) for Probands, Parents, and Controls (collapsed). [file 13229_2019_296_MOESM4_ESM.docx]
