# Supplementary material for: Familiality of behavioral flexibility and response inhibition deficits in autism spectrum disorder (ASD)
Source: Mol Autism. 2019 Dec 12;10:47. doi: 10.1186/s13229-019-0296-y (PMC6909569; doi:10.1186/s13229-019-0296-y)
Supplement: Supplementary file 5 — Additional file 5. Correlation Matrix of Relationship between BAP-Q scores and Primary Cognitive Control Variables for Parents of Individuals with ASD. [file 13229_2019_296_MOESM5_ESM.docx]

Additional file 5. Correlation Matrix of Relationship between BAP-Q scores and Primary Cognitive Control Variables for Parents of Individuals with ASD

|  | PRL Error Rate | SST Error Rate | SST RT Slowing |
| --- | --- | --- | --- |
| Aloof Score | -.104 | -.031 | -.013 |
| Pragmatic Score | -.114 | -.134 | -.146 |
| Rigid Score | -.207* | .034 | -.080 |
| Total Score | -.170 | -.045 | -.090 |

Z-scores used for all cognitive control variables
BAP-Q subscale and total scores
*p<.05
