# Supplementary material for: Familiality of behavioral flexibility and response inhibition deficits in autism spectrum disorder (ASD)
Source: Mol Autism. 2019 Dec 12;10:47. doi: 10.1186/s13229-019-0296-y (PMC6909569; doi:10.1186/s13229-019-0296-y)
Supplement: Supplementary file 6 — Additional file 6. Non-age adjusted findings. [file 13229_2019_296_MOESM6_ESM.docx]

Additional file 6. Non-age adjusted findings

For PRL error rate, probands, parents, proband controls, and parent controls differed (F=7.830, p<.001). That is, probands had higher PRL error rates than parents (t=3.710, p=.001) and parent controls (t=4.680, p <.001), but had similar PRL error rate to proband controls (t=2.376, p=.084). Parents had similar PRL error rates to proband controls (t=-.280, p=.992) and to parent controls (t=2.023, p=.182). Proband controls also had similar PRL error rates to parent controls (t=-1.715, p=.318). These findings do not substantively differ from those using age-adjusted variables.

For SST error rate, probands, parents, proband controls, and parent controls differed (F=32.289, p<.001). That is, probands had higher SST error rates than parents (t=8.696, p<.001), proband controls (t=3.014, p=.015) and parent controls (t=8.233, p <.001). Parents had lower SST error rates than proband controls (t=-3.319, p=.006), but similar error rates to parent controls (t=-1.818, p=.247). Proband controls had higher SST error rates than parent controls (t=-4.128, p<.001). Without adjusting for age, parents of individuals with ASD perform relatively similar to, if not better than, controls. Of note, probands had higher error rates than parents on both PRL and SST tasks, which was not found with our age-adjusted findings. This is an important as it indicates once age is taken into account, individuals with ASD and parents of individuals with ASD have similar levels of inhibitory control impairment.

For SST RT slowing, probands, parents, proband controls, and parent controls differed (F=54.301, p<.001). That is, probands had reduced SST RT slowing compared to than parents (t=-12.161, p<.001), proband controls (t=-3.746, p<.001) and parent controls (t=-8.749, p <.001). Parents demonstrated greater RT slowing than proband controls (t=5.171, p<.001), but a similar amount to parent controls (t=0.625, p=.553). Proband controls had reduced SST RT slowing than parent controls (t=-3.875, p<.001). These results differ slightly from those from age-adjusted analyses, as parents displayed similar RT slowing to the collapsed control group.
